# Effect of repeat catheter ablation vs. antiarrhythmic drug therapy among patients with recurrent atrial tachycardia/atrial fibrillation after atrial fibrillation catheter ablation: data from CHINA-AF registry

**DOI:** 10.1093/europace/euac169

**Published:** 2022-09-26

**Authors:** Lu Zhou, Liu He, Wei Wang, Changyi Li, Songnan Li, Ribo Tang, Caihua Sang, Nian Liu, Chenxi Jiang, Ronghui Yu, Deyong Long, Xin Du, Jianzeng Dong, Changsheng Ma

**Affiliations:** Department of Cardiology, Beijing AnZhen Hospital, Capital Medical University, National Clinical Research Centre for Cardiovascular Diseases, Beijing Advanced Innovation Center for Big Data-Based Precision Medicine for Cardiovascular Diseases, Beijing 100029, China; Department of Cardiology, Beijing AnZhen Hospital, Capital Medical University, National Clinical Research Centre for Cardiovascular Diseases, Beijing Advanced Innovation Center for Big Data-Based Precision Medicine for Cardiovascular Diseases, Beijing 100029, China; Department of Cardiology, Beijing AnZhen Hospital, Capital Medical University, National Clinical Research Centre for Cardiovascular Diseases, Beijing Advanced Innovation Center for Big Data-Based Precision Medicine for Cardiovascular Diseases, Beijing 100029, China; Department of Cardiology, Beijing AnZhen Hospital, Capital Medical University, National Clinical Research Centre for Cardiovascular Diseases, Beijing Advanced Innovation Center for Big Data-Based Precision Medicine for Cardiovascular Diseases, Beijing 100029, China; Department of Cardiology, Beijing AnZhen Hospital, Capital Medical University, National Clinical Research Centre for Cardiovascular Diseases, Beijing Advanced Innovation Center for Big Data-Based Precision Medicine for Cardiovascular Diseases, Beijing 100029, China; Department of Cardiology, Beijing AnZhen Hospital, Capital Medical University, National Clinical Research Centre for Cardiovascular Diseases, Beijing Advanced Innovation Center for Big Data-Based Precision Medicine for Cardiovascular Diseases, Beijing 100029, China; Department of Cardiology, Beijing AnZhen Hospital, Capital Medical University, National Clinical Research Centre for Cardiovascular Diseases, Beijing Advanced Innovation Center for Big Data-Based Precision Medicine for Cardiovascular Diseases, Beijing 100029, China; Department of Cardiology, Beijing AnZhen Hospital, Capital Medical University, National Clinical Research Centre for Cardiovascular Diseases, Beijing Advanced Innovation Center for Big Data-Based Precision Medicine for Cardiovascular Diseases, Beijing 100029, China; Department of Cardiology, Beijing AnZhen Hospital, Capital Medical University, National Clinical Research Centre for Cardiovascular Diseases, Beijing Advanced Innovation Center for Big Data-Based Precision Medicine for Cardiovascular Diseases, Beijing 100029, China; Department of Cardiology, Beijing AnZhen Hospital, Capital Medical University, National Clinical Research Centre for Cardiovascular Diseases, Beijing Advanced Innovation Center for Big Data-Based Precision Medicine for Cardiovascular Diseases, Beijing 100029, China; Department of Cardiology, Beijing AnZhen Hospital, Capital Medical University, National Clinical Research Centre for Cardiovascular Diseases, Beijing Advanced Innovation Center for Big Data-Based Precision Medicine for Cardiovascular Diseases, Beijing 100029, China; Department of Cardiology, Beijing AnZhen Hospital, Capital Medical University, National Clinical Research Centre for Cardiovascular Diseases, Beijing Advanced Innovation Center for Big Data-Based Precision Medicine for Cardiovascular Diseases, Beijing 100029, China; Heart Health Research Center (HHRC), Beijing 102206, China; The George Institute for Global Health, The University of New South Wales, Sydney 2052, Australia; Department of Cardiology, Beijing AnZhen Hospital, Capital Medical University, National Clinical Research Centre for Cardiovascular Diseases, Beijing Advanced Innovation Center for Big Data-Based Precision Medicine for Cardiovascular Diseases, Beijing 100029, China; Department of Cardiology, The First Affiliated Hospital of Zhengzhou University, Zhengzhou, Henan Province 450052, China; Department of Cardiology, Beijing AnZhen Hospital, Capital Medical University, National Clinical Research Centre for Cardiovascular Diseases, Beijing Advanced Innovation Center for Big Data-Based Precision Medicine for Cardiovascular Diseases, Beijing 100029, China

**Keywords:** Atrial fibrillation, Repeat ablation, Landmark analysis

## Abstract

**Aims:**

Although several studies have proved that repeat catheter ablation is beneficial to recurrent atrial tachycardia (AT)/atrial fibrillation (AF) after AF catheter ablation, the hard endpoints of the effect of catheter ablation on recurrent AT/AF patients after AF catheter ablation remains unclear. Our study aims to compare the effect of catheter ablation and drug therapy on recurrent AT/AF patients after AF catheter ablation.

**Methods and results:**

Four thousand nine hundred and thirteen consecutive patients with recurrent AT/AF after catheter ablation from the China-AF registry were enrolled. The patients were divided into two study groups: the repeat catheter ablation group and the medical therapy group. The primary endpoint is a composite of cardiovascular mortality or ischaemic stroke or major bleeding events. Secondary endpoints were each component of the primary endpoints and AF recurrence rate. Landmark analysis and Cox regression were used in the statistical analysis. We chose landmark 36 months as the primary landmark date. Over a median follow-up period of 40 ± 24 months, 4913 patients were divided into either the repeat ablation group or the medical therapy group. The cumulative incidence of the composite primary outcome was significantly lower in the repeat ablation group than the medical therapy group (adjusted hazard ratio = 0.56; 95% confidence interval: 0.35–0.89; *P* = 0.015) of landmark 36 months (2359 patients were included in medical therapy group and 704 patients were included in repeat ablation group at landmark 36 months). However, all secondary endpoints were not statistically different in the two groups, including cardiovascular mortality, ischaemic stroke, major bleeding events, and AF recurrence rate.

**Conclusion:**

Based on this research, in recurrent AT/AF patients after a catheter ablation procedure, compared with medical therapy, repeat catheter ablation may significantly reduce the risk of the endpoint of composite cardiovascular mortality, ischaemic stroke, and major bleeding events.

**What’s new?**

- This is the first article to evaluate the hard endpoints (cardiovascular death, ischaemic stroke, and major bleeding events) in patients with recurrent atrial tachycardia (AT)/atrial fibrillation (AF) after an AF catheter ablation procedure.
- This study has the largest sample size to date to focus on the prognosis of recurrent AT/AF patients after AF catheter ablation.
- The superiority of catheter ablation over medical therapy for recurrent AT/AF after AF ablation was found in the composite endpoint of cardiovascular mortality, ischaemic stroke, or major bleeding events.

## Introduction

Atrial fibrillation (AF) is the most common cardiac tachyarrhythmia, severely threatening the expectation and quality of life.^^1^,2^ Catheter ablation and antiarrhythmic drugs (AADs) are the most common rhythm control strategies. Antiarrhythmic drugs have been regarded as the first-line therapy for treating AF for many years, but recently, plenty of research comparing the rhythm control strategies has proved that catheter ablation has superiority in many aspects.^3–6^ Therefore, the AF guidelines recommended that catheter ablation should/may be considered as first-line rhythm control therapy to improve symptoms in selected symptomatic patients.^7^ Nowadays, a large number of people have received catheter ablation as the first-line option to treat AF. However, the success rate of catheter ablation stayed at ∼50–70% for an extended period of time.^4,5,8^ Moreover, this causes a new problem: which is the better way to treat recurrent AF, repeat ablation, or AAD therapy? A number of studies have already shown that compared with drug therapy, the patients who received repeat ablation have a lower rate of atrial tachycardia (AT)/AF recurrence.^8–10^ Few studies have compared the hard endpoints (hard endpoints, which are contradicted the surrogate endpoints are considered the backbone of the clinical trials) of repeat ablation and medical therapy in patients with recurrent AT/AF after catheter ablation. Our study aimed to compare the survival outcomes with the repeat ablation group and AAD group in patients with recurrent AT/AF after AF catheter ablation.

## Methods

### Registry information and clinical follow-up

China-AF registry is a prospective, multicentre, hospital-based registry of patients with AF recruited from 20 tertiary and 12 nontertiary hospitals in Beijing, China, where most AF patients in Beijing are included. The design and rationale of the China-AF registry have been described previously.^11^ Most patients enrolled were followed through telephone interviews, outpatient, and WeChat APP. Follow-up visits are at the third, sixth, and twelfth after treatment and every 6 months thereafter. Approval was obtained from all participating institutions’ ethic boards, and written informed consent was acquired from each patient. Study management was arranged and coordinated by Beijing Anzhen Hospital. Follow-up information was recorded in the registry database.

### Study population

Consecutive AF patients with age >18-year-old who experienced recurrent AT/AF after a 3-month blanking period since the first catheter ablation procedure between August 2011 and September 2020 were enrolled from the China-AF registry. The exclusion criteria were: (i) patients whose last follow-up date was still in the blanking period. (ii) Patients who contradicted anticoagulated drugs.

Eligible patients were enrolled when they documented recurrent AT/AF (>30 s AT/AF after the blanking period) after the blanking period of an AF catheter ablation. Three methods were performed to monitor AF recurrence: (i) routinely follow-up examination: electrocardiogram (ECG) and 24 h-Holter were performed every month in the first 3 months and every 6 months thereafter; (ii) symptom suggested examination: additional ECGs would be performed if the patient experienced tachycardia symptoms; (iii) opportunistic examination: ECGs or HOLTERs recorded for manual examinations or other diseases were involved. The imagines of each exam were transmitted through WeChat APP to Beijing Anzhen Hospital and were diagnosed by cardiologists.

### Study outcomes

The primary endpoint is a composite of the first occurrence of cardiovascular mortality or ischaemic stroke or major bleeding events. Secondary endpoints were each component of the primary endpoints and AF recurrence rate.

### Landmark analysis

Analysis of the outcomes of recurrent AF patients with repeat catheter ablation by using the observational registry data was complicated by several problems. First, the dates of repeat ablation in these patients were not relevant to cohort entry dates, so the time-to-treatment bias could not be ignored. Second, some patients may have had an outcome before they underwent repeat catheter ablation. To overcome these problems, the landmark analysis was used in our study, which is designed to minimize immortal time bias and reverse causality efficiency.^12–14^ In a landmark method, only the patients who do not have outcomes before the landmark time could be included in the analysis, and patients were grouped according to interventions that were taken between baseline date and landmark time.^12^ This method is capable of applying to any study that does not match the equal proportion risk model, and also can diminish the survival bias, such as immortal time bias.

The landmark time would be selected as a specific time after the cohort entry date, so in our study, 36 months after the study entry date was chosen as the primary landmark date to guarantee an ample size of the repeat ablation group. The patients who had occurred the primary endpoint events before the landmark time were excluded from the analysis, and those underwent a repeat catheter ablation between cohort entry date and landmark date were regarded as exposed, while other were regarded as unexposed.

Based on whether the patient exposed or not, the enrolled patients were divided into one of the two study groups: repeat catheter ablation group or medical therapy group.

### Ablation procedures

The index ablation procedures were all guided by a three-dimensional electroanatomic mapping system (CARTO3 system).^15^ Patients basically underwent circumferential pulmonary vein isolation (PVI). For paroxysmal AF patients, cavotricuspid isthmus (CTI) linear ablation would be performed if the paroxysmal AF patients were diagnosed with typical AFL before the procedure. And other additional ablation strategies include the ablation of the left atrial (LA) roof, and mitral isthmus (MI) were seldomly performed in our registry; for persistent AF, linear ablations across the LA roof, MI, and CTI were systematically applied in addition to PVI (‘2C3L’ strategy).^16^ Cardioversion was performed if AF persisted or converted to an organized atrial tachyarrhythmia (OAT) following the initial PV antrum and linear ablations. If cardioversion failed or AF immediately relapsed, an intravenous infusion of amiodarone was administered before repeat cardioversion.

The repeat ablation procedure followed the ‘2C3L’ strategy where conduction gaps were targeted and clinical OATs were also mapped and ablated; however, CFAEs ablation was not performed during the repeat procedure. After complete PVAI and blockage across the three lines (LA roof, MI, and CTI) were achieved, burst pacing to pace at 200 ms was applied from proximal CS to induce tachycardia.

### Anticoagulant therapy

All patients whose CHA_2_DS_2_-VASc score ≥2 in men and ≥3 in women would receive anticoagulant therapy for the first time when they are diagnosed with AF. Novel oral anticoagulants (NOACs) would be the first option for these patients, while others who cannot tolerate NOACs would be treated with vitamin-k antagonist according to the guidelines of AF. The doses of each drug would be directed strictly by the guideline as well. Novel oral anticoagulants would be stopped to use in the ablation arm prior 1–2 days to the ablation procedure. Unfractionated heparin was used in the ablation procedure to maintain the activated clotting time to 300–350 s. After the procedure, we suggested the patients use NOACs during the whole blanking period. Furthermore, the patients’ anticoagulant therapy would be assessed and directed by an electro cardiologist after the blanking period, based on the guideline of AF.

### Antiarrhythmic therapy

Antiarrhythmic drugs were used routinely in the necessity patients according to the guidelines of AF, as well as the doses of these drugs. Commonly, amiodarone was used as the first choice in persistent AF patients otherwise they have structural heart disease while Class Ic AADs was applied to the paroxysmal AF patients with priority. In the repeat ablation group, antiarrhythmic therapy would be recommended during the blanking period. After the blanking period, whether the patient should utilize the AADs was assessed by a cardiovascular expert in Beijing Anzhen Hospital.

### Examination

In the ablation group, transthoracic echocardiography was performed to assess the structure of the atrial and ventricle, such as left ventricular ejection fraction (LVEF) and LA diameter. At the same time, transoesophageal echocardiography was also performed to acknowledge the absence of LA/LA appendage thrombus before the ablation procedure.

### Statistics analysis

Continuous variables were presented as median plus interquartile range, and categorical variables were presented as mean and standard deviation. Baseline characteristics between repeat catheter ablation group and AAD group were assessed with *χ*^2^ test or Wilcoxon test. Clinical outcomes were compared between the two groups using landmark analysis at 36 months after study entry. The Kaplan–Meier estimator was used to calculate the time to event-rate of the primary and secondary endpoints, whereas log-rank tests were applied to compare incidences of the study endpoints. Hazard ratios (HRs) and their 95% confidence intervals (CIs) were calculated to demonstrate the associations of repeat catheter ablation with the clinical outcomes, using Cox proportional-hazards regression models, adjusted for age, gender, sinus rhythm maintenance duration before AF recurrent, type of AF, body mass index, smoking status, coronary artery disease, hypertension, heart failure, previous bleeding, previous stroke/TIA, estimated glomerular filtration rate (eGFR), LA diameter, left ventricular end-diastolic diameter (LVEDD), LVEF, as well as time-varying covariates (use of β blocker, calcium channel blocker (CCB), digoxin, propafenone, amiodarone, and sotalol during follow-ups). We also performed sensitivity analyses. We examined the effects of repeat ablation at the landmark time of 12, 24 months. All statistical comparisons were two-sided, and *P*-values < 0.05 were considered statistically significant. Analyses were performed using the SAS software version 9.4 (SAS Institution Inc., Cary, NC, USA).

## Result

### Baseline characteristic

A total of 4913 patients who recurrent AT/AF in AF catheter ablation patients enrolled in this cohort with a mean follow-up of 40 ±24 months, 1230 of them underwent a repeat ablation. Of the entire cohort participants, 143 (49.8%) had one of the fixed outcome events prior to the landmark time and were excluded from further analyses. Those excluded were anticipated to be more likely to experience cardiovascular disease but whose baseline character was similar to the rest of the cohort participants. The baseline characteristics are shown in *Table 1*.

**Table 1:** Baseline characteristic (grouped by landmark 36 months)

	Repeat ablation group	Medical therapy group	*P*-value
Age (years, mean ± SD)	61.4 ± 10.8	60.4 ± 10.7	<0.001
Sinus rhythm maintenance duration before AF recurrent [months, median (Q1, Q3)]	14(4, 33)	9(3, 16)	<0.001
Gender			0.664
ȃFemale (%)	36.3	35.7	
Body mass index [kg/m^2^, median(Q1, Q3)]	24.8(23.5, 27.3)	25.4(23.5, 27.8)	<0.001
AF severity (%)			<0.001
ȃI (least severe)	9.9	5.8	
ȃII	62.5	64.1	
ȃIII (most severe)	27.6	30.1	
Type of AF (%)			0.002
ȃPersistent	39.0	34.6	
NYHA class (%)			<0.001
ȃI	60.2	73.9	
ȃII	33.0	20.1	
ȃIII	5.5	5.4	
ȃIV	1.3	0.6	
CHA2DS2-VASc score, median(Q1, Q3)	2(1, 3)	2(1, 3)	0.112
HAS-BLED score, median(Q1, Q3)	1(1, 2)	1(1, 2)	0.004
Current smoking (%)	10.3	13.7	0.004
LA size [mm, median(Q1, Q3)]	38(36.2, 42.7)	40(36.0, 43.0)	0.009
LVEDd [mm, median(Q1, Q3)]	46(45, 50)	48(45, 51)	<0.001
EF [mm, median(Q1, Q3)]	60(60, 66)	64(60, 68)	<0.001
Anticoagulant (%)	95.8	95.5	0.662
Antiarrhythmic drugs (%)	21.1	22.8	0.346
Antiplatelet	37.8	33.7	0.055
Major bleeding event (%)	3.20	5.06	0.002
Stroke/TIA (%)	11.7	11.5	0.853
Diabetes (%)	22.3	20.3	0.093
Hypertension (%)	55.7	58.7	0.043
CAD (%)	13.1	11.4	0.090
Heart failure (%)	6.8	5.5	0.068
AAD	38.3	57.9	<0.001
Β blocker	17.2	34.0	<0.001
CCB	1.4	3.0	<0.001
Digoxin	0.46	1.20	0.008
Propafenone	12.3	24.1	<0.001
Amiodarone	18.7	33.0	<0.001
Sotalol	0.69	1.55	0.010

The patients in the repeat ablation group had a mean age of 61.4 years, 36.3% were women, and 39% were diagnosed as persistent AF. While those patients in the medical therapy group had a mean age of 60.4 years, 35.7% were women, and 35% were diagnosed as persistent AF. The account of persistent AF patients was higher in patients of repeat ablation group (42% vs. 35%, *P* < 0.001), as well as median age (61 vs. 60 years; *P* < 0.001). Compared with the medical therapy group, patients in the repeat ablation group had a higher risk of hypertension and priory major bleeding events and were more likely to have a lower HAS-BLED score, as well as larger LVEDD and LVEF, while showed no statistically different comorbidities in prior ischaemic stroke, diabetes mellitus, coronary artery disease, and heart failure. The difference of LA size, CHA_2_DS_2_-VASc scores, and the utilization of anticoagulated drugs, AADs, and antiplatelet drugs between two groups was of no statistical significance. The percentage of NYHA Class I was lower in the repeat ablation group than medical therapy group.

The rate of the baseline utilization of β blocker, CCB, digoxin, propafenone, amiodarone, sotalol between these two groups was comparable.

## Primary outcome

**Figure 1:**
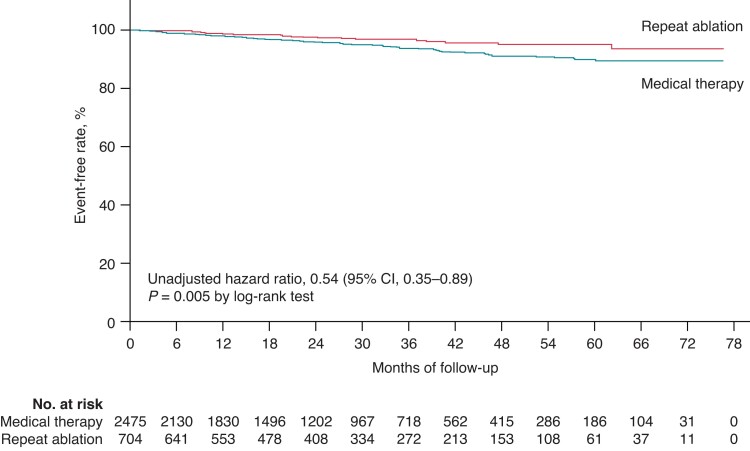
Kaplan–Meier estimates of primary outcome analysis.

Twenty-one (1.15%) primary outcome events occurred in the repeat ablation group and 120 (2.13%) occurred in the medical therapy group during the follow-up period. The Kaplan–Meier curve of the primary outcome is shown in *Figure 1*. The cumulative incidence of the composite primary outcome was significantly lower in the repeat ablation group than the medical therapy group (unadjusted HR = 0.54; 95% CI: 0.34–0.86; *P* = 0.009).

*Table 2* shows the incidence rates and the primary endpoint in the univariable analysis and multivariable analysis of landmark 36 months. According to multivariable Cox regression, patients who underwent repeat catheter ablation had a significant lower risk of the primary outcome than those receiving medical therapy (adjusted HR = 0.56; 95% CI: 0.35–0.89; *P* = 0.015).

**Table 2:** The incidence rates and primary and the secondary endpoints in the univariable and multivariable analysis of landmark 36 months, in multivariable cox analysis, we adjusted for age, gender, sinus rhythm maintenance duration before AF recurrent, type of AF, BMI, smoking status, coronary artery disease, hypertension, heart failure, previous bleeding, previous stroke/TIA, eGFR, left atrial diameter, LVEDD, LVEF and use of β blocker, CCB, digoxin, propafenone, amiodarone, sotalol were included as time-varying covariates

	Incidence rate (%)		Unadjusted HR (95% CI)	*P*	Adjusted HR (95% CI)	*P*
	Repeat ablation	Medical therapy				
Primary endpoint	1.15	2.13	0.54(0.35, 0.89)	0.009	0.56(0.35, 0.89)	0.015
Cardiovascular death	0.05	0.30	0.17(0.03, 1.49)	0.086	0.19(0.03, 1.46)	0.111
Ischaemic stroke	0.86	1.36	0.63(0.38, 1.11)	0.090	0.64(0.37, 1.10)	0.102
Major bleeding events	0.26	0.67	0.40(0.17, 1.13)	0.055	0.41(0.16, 1.06)	0.066
AF recurrence	13.39	19.58	0.74(0.54, 1.06)	0.065	0.75(0.53, 1.05)	0.089

## Secondary outcomes

There were five major bleeding events in the repeat ablation group and 39 cases in the medical therapy group, with rates of 0.26% and 0.67%, though the difference was not statistically significant, a higher risk of major bleeding in medical therapy group was indicated (adjusted HR = 0.41; 95% CI: 0.16–1.06, *P* = 0.066). In addition, other secondary endpoints, including ischaemic stroke and all-cause mortality, were not statistically different in the two groups.

**Figure 2:**
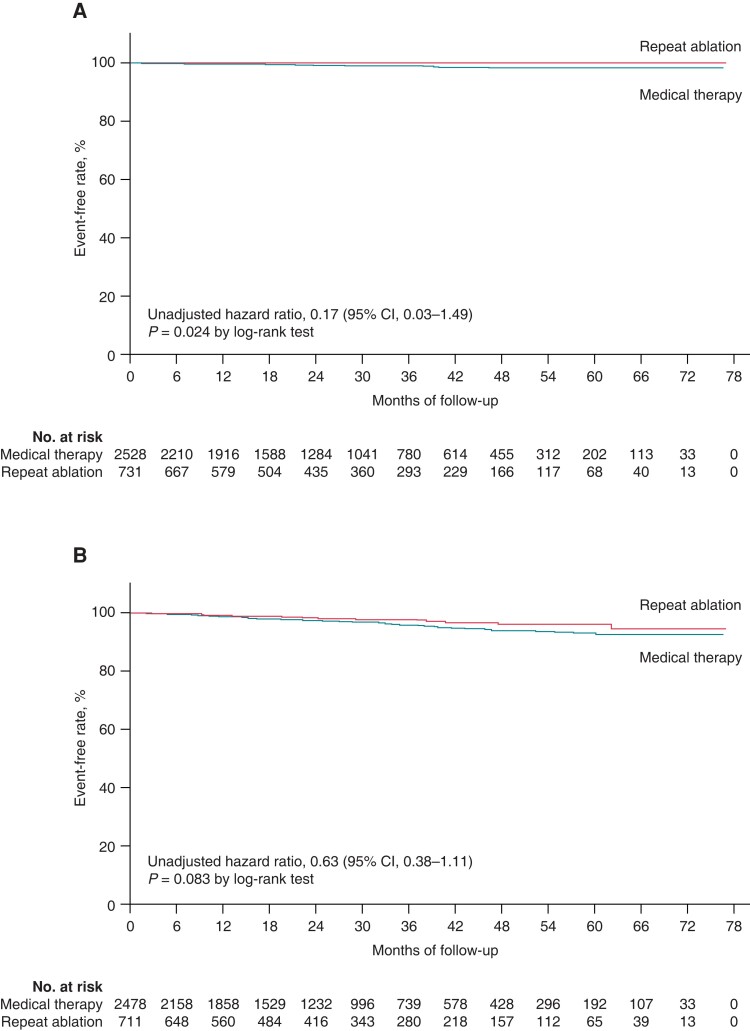

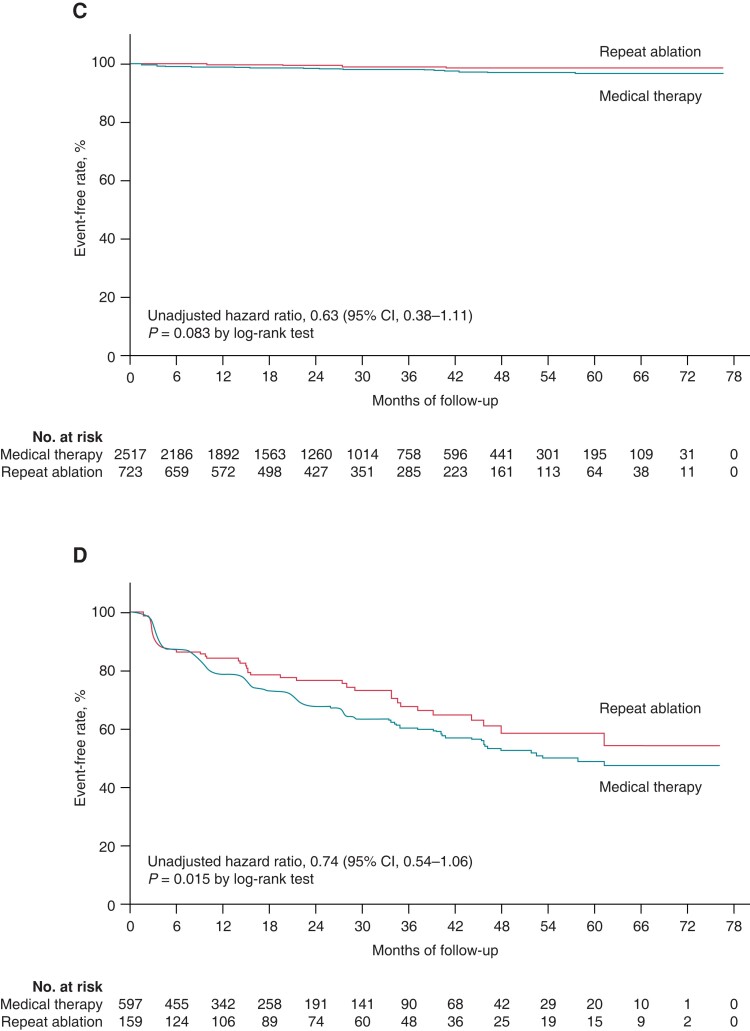
(*A*) Kaplan–Meier estimates of the cardiovascular deaths analysis. (*B*) Kaplan–Meier estimates of the ischaemic stroke analysis. (*C*) Kaplan–Meier estimates of the major bleeding events analysis. (*D*) Kaplan–Meier estimates of the AF recurrent analysis.

*Figure 2* shows the Kaplan–Meier estimator in each secondary endpoints of the landmark 36 months. The incidence rates and the secondary endpoints in the univariable and multivariable analysis of landmark 36 months were shown in *Table 2*.

## Atrial fibrillation recurrence rate

There were 906 (91.2%) patients undergoing one repeat ablation while 90 (8.15%) patients with a third ablation and 7 (0.7%) with a fourth ablation after the second one. *Figure 2D* shows the AF recurrence rate for repeat ablations since the landmark of 36 months. The AF recurrence rate had a lower trend in the repeat ablation group than the medical therapy group (adjusted HR = 0.75, 95% CI: 0.53–1.05, *P* = 0.089) (*Table 2*).

## Sensitivity analysis

Similar analysis was also performed for landmark of 12 and 24 months. The percentage of repeat ablation group patients included in the landmark analysis are lower for landmark of 12 months (4218 patients were included in medical therapy group and 604 patients were included in repeat ablation group at landmark 12 months) and for landmark of 24 months (3115 patients were included in medical therapy group and 696 patients were included in repeat ablation group at landmark 24 months). In *Table 3*, the primary outcome event was lower in the repeated ablation group of landmark 12 months (adjusted HR = 0.70, 95% CI: 0.48–1.03, *P* = 0.068) and landmark 24 months (adjusted HR = 0.79, 95% CI: 0.54–1.15, *P* = 0.218), but the differences between groups were not statistically significant. In addition, the repeat ablation group had a significant lower risk of ischaemic stroke of landmark 12 months (adjusted HR = 0.54, 95% CI: 0.32–0.90, *P* = 0.019), as well as a significant lower risk of cardiovascular death of landmark 24 months (adjusted HR = 0.24, 95% CI: 0.06–0.99, *P* = 0.049). Other secondary endpoints of landmark 12 months and landmark 24 months, however, were not statistically different between the two groups. Besides, the repeat ablation group had a significantly lower rate of AF recurrence compared with the medical therapy group at landmark 12 months (adjusted HR = 0.70, 95% CI: 0.63–0.77, *P* < 0.001) and landmark 24 months (adjusted HR = 0.68, 95% CI: 0.54–0.85, *P* < 0.001) (*Table 3*).

**Table 3:** The primary and the secondary endpoints in the univariable and multivariable analysis of landmark 12 months and landmark 24 months, in multivariable cox analysis, we adjusted for age, gender, sinus rhythm maintenance duration before AF recurrent, type of AF, BMI, smoking status, coronary artery disease, hypertension, heart failure, previous bleeding, previous stroke/TIA, eGFR, left atrial diameter, LVEDD, LVEF and use of β blocker, CCB, digoxin, propafenone, amiodarone, and sotalol were included as time-varying covariates

	Landmark 12 months				Landmark 24 months			
	Unadjusted HR (95% CI)	*P*	AdjustedHR (95% CI)	*P*	Unadjusted HR (95% CI)	*P*	Adjusted HR (95% CI)	*P*
Primary endpoint	0.71(0.48, 1.03)	0.073	0.70(0.48, 1.03)	0.068	0.75(0.53, 1.13)	0.132	0.79(0.54, 1.15)	0.218
Cardiovascular death	0.70(0.28, 1.76)	0.442	0.67(0.27, 1.71)	0.406	0.24(0.06, 1.05)	0.048	0.24(0.06, 0.99)	0.049
Ischaemic stroke	0.56(0.33, 0.93)	0.025	0.54(0.32, 0,90)	0.019	0.77(0.50, 1.29)	0.283	0.79(0.49, 1.27)	0.329
Major bleeding events	0.95(0.49, 1.85)	0.874	0.96(0.49, 1.88)	0.907	0.69(0.36, 1.52)	0.341	0.74(0.36, 1.51)	0.402
AF recurrence	0.70(0.63, 0.77)	<0.001	0.70(0.63, 0.77)	<0.001	0.71(0.54, 0.86)	0.002	0.68(0.54, 0.85)	0.001

## Discussion

This is a prospective, multicentre, real-world cohort study. It was found that repeat ablation for recurrent AF patients was associated with a significantly lower risk of the composite outcome of the first occurrence of cardiovascular death, ischaemic stroke, or major bleeding event than medical therapy. Besides, among the recurrent AF patients, the one who had undergone repeat ablation had a lower trend of major bleeding events risk.

Several studies have already reported the benefit of first-time catheter ablation in hard endpoints. In Castle-AF (Catheter Ablation for Atrial Fibrillation with Heart Failure) trial,^5^ researchers reported that among AF patients with heart failure, compared with medical therapy, catheter ablation can significantly reduce the risk of a composite endpoint of death or hospitalization for worsening heart failure, as well as all-cause mortality and cardiovascular death. While In CABANA (Effect of Catheter Ablation vs. Antiarrhythmic Drug Therapy on Mortality, Stroke, Bleeding, and Cardiac Arrest Among Patients With Atrial Fibrillation trial) trial,^4^ data have shown no statistically difference neither in the composite endpoint of death, disabling stroke, serious bleeding, or cardiac arrest nor other hard endpoints such as all-cause mortality, cardiovascular hospitalization. However, it only proved that catheter ablation is associated with a significantly lower risk of AF recurrence. As for real-world data, the result from Swedish health registries showed the superiority of catheter ablation in ischaemic stroke, all-cause mortality, and cardiovascular death.^6^ On the contrary, the ORBIT-AF (Outcomes Registry for Better Informed Treatment of Atrial Fibrillation) trail reported the adjusted risk of death, cardiovascular hospitalization and the composite endpoint of death, stroke/TIA, and CHF were similar in both groups. Patients with incident AF ablation had higher risk of cardiovascular hospitalization than the patients who did not undergo catheter ablation.^3^ For instance, whether the initial ablation could improve the hard endpoints in AF patients is still controversial. Besides, the results of repeat ablation were seldom reported, and the information on the impact of repeat ablation on the hard endpoints was limited.

Recently, some soft endpoints have been reported with catheter ablation in patients who received repeat catheter ablation procedures. A randomized control trial (RCT) illustrated the superiority of catheter ablation over medical therapy for recurrent AT after persistent AF ablation in outcomes like sinus rhythm maintenance, long-term safety, and improved quality of life.^8^ A multicentre, prospective cohort study that recruited 1404 AF patients who underwent catheter ablation confirmed that repeat ablation could achieve a higher success rate.^17^ A prospective cohort study based on GERMAIN ABLATION REGISTRY gives evidence of the improvement in the quality of life with repeat ablation. However, it also showed no significant differences in mortality, major adverse cardiovascular events, major adverse cardiac and cerebrovascular events, and composite safety endpoint between repeat ablation group and index ablation group during 1-year follow-up.^10^ Furthermore, the ORBIT-AF (Outcomes Registry for Better Informed Treatment of Atrial Fibrillation) study demonstrates that incident AF ablation had a lower rate of all-cause and cardiovascular death, but these differences did not reach statistical significance.^3^ Our study, which differ from those previous trials, evaluated the hard endpoints of the repeat ablation procedure. As a large real-world study, we selected a composite of cardiovascular death, ischaemic stroke and major bleeding events as our primary endpoint in parallel with the previous influential RCTs. Drawing valuable lessons from the CABANA and Castle-AF trial, we followed the patients for more than 3 years to acquire a more convincing result. This is the first study to focus on hard endpoints of repeat ablation procedure, showing superior with repeat ablation in the composite of cardiovascular death, ischaemic stroke, major bleeding event. A landmark analysis was used to reduce the time-to-treatment bias and to ensure that there were enough patients in the repeat ablation group. Landmark of 36 months was chosen as the landmark time for the reasons stated above. Similar HR of landmark 12 months and 24 months, which showed superior of repeat catheter ablation to landmark 36 months were also obtained, but the differences were not statistically significant. We think the results of landmark 12 months and 24 months were not statistically different between the two groups is mainly due to the number of patients and endpoint events in repeat catheter ablation group of landmark 12 months and 24 months were lower than landmark 36 months, which may have contributed to a reduction in statistical power.

On the other hand, there was no significant difference in sinus rhythm rate between the ablation and medical therapy groups. We speculated that this was mainly due to the patients who maintained sinus rhythm often regarded themselves as healthy people and neglected to submit ECG or HOLTER recordings to the follow-up staff, which may resulted in some missing data.

In summary, this study may provide new evidence to treat repeat ablation procedures as first-line rhythm control therapy.

## Limitation

There are some limitations: First, in a real-world study, which was not randomized, there may be some unknown confounding factors, which may influence the accuracy of the study results. Second, the study was conducted between August 2011 and September 2020, the ablation technics and facilities were developed inordinately. The earlier ablation patients and later ones may use different technic. Third, owing to the date of repeat ablation independent of the study entry date, we use a landmark analysis and sensitivity analysis, but the immortal time bias could still exist.

## Conclusion

Based on this research, in recurrent AT/AF patients after a catheter ablation procedure, compared with medical therapy, repeat catheter ablation may significantly reduce the risk of the endpoint of composite cardiovascular mortality, ischaemic stroke, and major bleeding events.

## Supplementary Material

euac169_Supplementary_Data

## Data Availability

The data underlying this article could not be shared publicly due to the privacy of individuals who enrolled in the registry. The data will be shared on reasonable request to the corresponding author.
