# Supplementary material for: Effect of repeat catheter ablation vs. antiarrhythmic drug therapy among patients with recurrent atrial tachycardia/atrial fibrillation after atrial fibrillation catheter ablation: data from CHINA-AF registry
Source: Europace. 2022 Sep 26;25(2):382–9. doi: 10.1093/europace/euac169 (PMC10103561; doi:10.1093/europace/euac169)
Supplement: euac169_Supplementary_Data [file euac169_supplementary_data.docx]

|  | Hazard ratio | *P* |
| --- | --- | --- |
| Repeat catheter ablation | 0.56 | 0.015 |
| age | 1.02 | 0.064 |
| female | 1.66 | 0.010 |
| First AF recurrence duration | 1.01 | 0.503 |
| BMI | 0,95 | 0.048 |
| LA | 1.05 | 0.007 |
| LVEDD | 1.00 | 0.993 |
| LVEF | 0.99 | 0.524 |
| Persistent AF | 0.96 | 0.830 |
| eGFR | 1.74 | 0.062 |
| Current smoking | 1.74 | 0.032 |
| CAD | 1.10 | 0.697 |
| HF | 1.51 | 0.144 |
| Stroke/TIA | 1.74 | 0.008 |
| Bleeding events | 0.64 | 0.281 |
| Hypertension | 1.77 | 0.007 |
| diabetes | 1.90 | <0.001 |
| β blocker | 0.93 | 0.666 |
| CCB | 0.42 | 0.384 |
| Digoxin | 0.97 | 0.943 |
| Propafenone | 1.46 | 0.162 |
| amiodarone | 1.08 | 0.804 |
| sotalol | 0.92 | 0.881 |
| Table S1 The results of the multivariable Cox regression for all covariates for the primary endpoint（composite of the first occurrence of cardiovascular mortality or ischemic stroke or major bleeding events）. In multivariable cox analysis, we adjusted for age, gender, Sinus rhythm maintenance duration before AF recurrent, type of AF, BMI, smoking status, coronary artery disease, hypertension, heart failure, previous bleeding, previous stroke/TIA, EGFR, left atrial diameter, LVEDD, LVEF and use of β blocker, CCB, Digoxin, Propafenone, amiodarone and sotalol were included as time-varying covariates. | | |
